# Preoperative incentive spirometry for preventing postoperative pulmonary complications in patients undergoing coronary artery bypass graft surgery: a prospective, randomized controlled trial

**DOI:** 10.1186/s13019-021-01628-2

**Published:** 2021-08-24

**Authors:** Essa M. Sweity, Aidah A. Alkaissi, Wafiq Othman, Ahmad Salahat

**Affiliations:** 1 Faculty of Graduate Studies, An-Najah National University, Nablus, 44839 Palestine; 2 Cardiology Department, An-Najah National University Hospital, Nablus, 44839 Palestine; 3 Department of Anaesthesiology and Intensive Care, Faculty of Medicine and Health Sciences, Nursing and Midwifery Department, An-Najah National University, Nablus, 44839 Palestine; 4 An-Najah National University Hospital, Nablus, 44839 Palestine

**Keywords:** Incentive spirometry, Postoperative pulmonary complications, Atelectasis, Oxygenation, Ventilation time, Coronary artery bypass grafting, CABG, Length of stay

## Abstract

**Background:**

Postoperative pulmonary complications (PPCs) often occur after cardiac operations and are a leading cause of morbidity, inhibit oxygenation, and increase hospital length of stay and mortality. Although clinical evidence for PPCs prevention is often unclear and crucial, measures occur to reduce PPCs. One device usually used for this reason is incentive spirometry (IS). The aim of the study is to evaluate the effect of preoperative incentive spirometry to prevent postoperative pulmonary complications, improve postoperative oxygenation, and decrease hospital stay following coronary artery bypass graft (CABG) surgery patients.

**Methods:**

This was a clinical randomized prospective study. A total of 80 patients were selected as candidates for CABG at An-Najah National University Hospital, Nablus-Palestine. Patients had been randomly assigned into two groups: incentive spirometry group (IS), SI performed before surgery (study group) and control group, preoperative spirometry was not performed. The 40 patients in each group received the same protocol of anesthesia and ventilation in the operating room.

**Result:**

The study findings showed a significant difference between the IS and control groups in the incidence of postoperative atelectasis. There were 8 patients (20.0%) in IS group and 17 patients (42.5%) in the control group (*p* = 0.03). Mechanical ventilation duration was significantly less in IS group. The median was four hours versus six hours in the control group (*p* < 0.001). Hospital length of stay was significantly less in IS group, and the median was six days versus seven days in the control group (*p* < 0.001). The median of the amount of arterial blood oxygen and oxygen saturation was significantly improved in the IS group (*p* < 0.005).

**Conclusion:**

Preoperative incentive spirometry for two days along with the exercise of deep breathing, encouraged coughing, and early ambulation following CABG are in connection with prevention and decreased incidence of atelectasis, hospital stay, mechanical ventilation duration and improved postoperative oxygenation with better pain control. A difference that can be considered both significant and clinically relevant.

*Trial registration* Thai Clinical Trials Registry: TCTR20201020005. Registered 17 October 2020—retrospectively registered.

## Introduction

Coronary artery disease (CAD) is the leading cause of death and disability worldwide [1]. Therefore, coronary artery bypass grafting (CABG) is indicated for patients with angina and suitable coronary anatomy, especially those with stenosis of the left main coronary artery or patients with the multivessel disease [2].

Postoperative pulmonary complications (PPCs) are a frequent incident following cardiac, thoracic, and abdominal surgeries [3]. PPCs are widespread following CABG surgery, and the incidence is between 30 and 60% [4]. PPC complications contribute significantly to morbidity, mortality, and hospitalization costs [5]. These complications include atelectasis, pulmonary infections such as pneumonia and bronchitis, pleural effusion, pulmonary edema, and respiratory insufficiency [6].

Atelectasis is a highly prevalent complication following coronary artery bypass graft (CABG) surgery [7]. There is no clear cause yet for atelectasis, but several factors may contribute, such as impairment in the function of the diaphragm, general anaesthesia ‘abdominal distension, chest wall shift, pain, and pleural effusions [8].

The pain and postoperative fear associated with changes in lung mechanics resulting from the surgery affect the performance of periodic deep inspiration and effective cough, allowing the accumulation of secretion, alveolar collapse, and changes in gas exchange [9].

Shaban et al. [10] evaluated the effect of respiratory exercise in acute respiratory complications and the length of time patient hospitalization undergoing coronary artery bypass surgery by video teaching pre-and postoperatively and revealed that the significant difference in the incidence rate of atelectasis decreased, concurrent with decreased hospital length of stay. Moreover, Oshvandi et al. [11] and Yánez-Brage et al. [12] reported that the preoperative respiratory exercises include deep breathing, effective coughing and use, motivational incentive spirometry (incentive spirometry, deep-breathing exercises, assisted coughing and early ambulation) compared to normal performance and the routine performed in the hospital, in reduction atelectasis is more effective after coronary artery bypass graft surgery. In contrast, Freitas et al. revealed that there is no benefit of IS decreasing PPCs in patients following CABG surgery [13].

Although clinical evidence regarding PPC prevention is often unclear, crucial measures occur to reduce PPCs. These include carefully individualized strategies for preventing atelectasis and aspiration of oral secretions, increasing the patient’s ability to mobilize, expectorate secretions and restore functional residual capacity [14]. In addition, several measures are applied to prevent PPCs, such as deep-breathing exercises, that IS, early ambulation, and positive airway pressure [15, 16].

Incentive spirometry (IS) is one tool frequently used for this purpose [3]. The IS is a handheld device used postoperative to reach effective inspiration. Patients practicing IS provide visual and positive feedback after inhaling at a determined flow volume rate and holding the inflation for at least 3 s [17]. IS intended to mimic normal sighing or yawning by supporting the patient to take long and slow deep breaths. This reduces pleural tension, supporting enhanced lung expansibility and improving ventilation-perfusion. In addition, atelectasis may be prevented or reversed when the procedure is repeated regularly [18].

IS was found to decrease the incidence of PPCs and length of stay after upper abdominal surgery [19]. By contrast, many study publications have questioned its effectiveness [20, 21].

Monitoring, instruction and teaching the patient how to use the IS are the responsibility of nursing and respiratory therapy staff. Respiratory therapy that involves periods of IS each day in addition to deep-breathing applications, guided coughing, early mobilization, and pain control can reduce the incidence of PPCs [22]. In addition, incentive spirometry may prevent PPCs in patients following CABG surgery [12].

Applications of deep breathing are shown to reduce the occurrence and severity of PPCs, such as pneumonia and atelectasis. Through application instruction, the nurse clarifies and displays how to take a deep and gradual breath and exhale gradually, three to five times every 1–2 h. Patients who carried out deep-breathing exercises had improved pulmonary function in contrast with nonpracticing groups [23].

Afrasiabi et al. conducted a study about the influence of IS on the oxygenation status of arterial blood gases following a CABG operation. Throughout six h after Extubation, the patient was handled, IS and preoperative, one h, and seven h after Extubation with arterial blood gases [R] obtained. The researcher revealed that there was no significant benefit in oxygenation status measured by ABG's after using IS [24]. Freitas et al., Carvalho et al., Eltorai et al., and Overend et al. have declared that, to date, there is no evidence to support the practice of IS to decrease PPCs. Although IS is still usually requested to reduce PPCs, despite the narrow evidence to support its advantages and the absence of a harmonized protocol, and they recommend that additional research is necessary to clarify this issue [13, 20, 21, 25]. Agostini and Singh differ from this opinion and have stated that this practice can improve pulmonary function [26].

Preoperative education gives health-related information for patients, which prepares them for surgery and helps to decrease the development of PPCs [27]. In numerous studies, it is suggested that postoperative incentive spirometry is practiced to decrease PPCs and decrease the length of stay (LOS), but the success of postoperative incentive spirometry is dependent not only on the postoperative, but also the preoperative period, which has been shown to improve oxygenation, decrease the incidence of PPCs and to decrease hospital LOS [28, 29]. and agreed with them Moradyan et al. [30] who revealed that receiving planned breathing exercises, including deep-breathing exercises, incentive spirometry and directed cough manoeuvres have better oxygenation after coronary artery bypass surgery. Another study has shown that the rate of pneumonia and atelectasis reduced with breathing exercise and IS in obese patients prior to CABG surgery [31]. By contrast, Moradian et al. [32] revealed that preoperative breathing exercise with incentive spirometry does not reduce pulmonary complications and Hypoxemia. IS training before and after the operation significantly improved lung inspiratory capacity and arterial oxygenation in CABG patients [33].

Since PPCs exhibit elevated rates of hospital costs, morbidity, mortality, and increased length of hospital stay following CABG surgery, it is evident that it is essential to discuss the use of IS preoperatively to reduce PPCs and to decrease postoperative length of stay in the intensive coronary care unit (ICCU) and in the hospital. The study aims to evaluate the effect of preoperative incentive spirometry in preventing postoperative pulmonary complications, improving postoperative oxygenation, and decreasing the length of stay at the hospital in patients following CABG surgery.

## Materials and methods

### Study design

The study was conducted as prospective cohort, randomized controlled trial (RCT). This design was adopted due the strength of the hierarchy of scientific evidence, namely, reduced bias and more accurate results.

#### Study setting and population

The study was conducted at AN Najah National University Hospital. Data was collected from CCU and Intermediate CCU wards. An-Najah National University Hospital has 200 beds, 5 beds for CCU and 16 beds for Intermediate CCU. It is a non-profit hospital, located in the Northern West Bank, Palestine.

Participants are adult patients scheduled for coronary artery bypass surgery, aged 18 or older, and patients who were well motivated and compliant.

### Sample and sampling

To investigate the optimal sample magnitude for the trial that safeguards an adequate effect to identify statistical significance, the effect of the trial was estimated at 80 percent power, with alpha levels at (*p* ≤ 0.05). Sample magnitude was computed as 37 patients for each group by using a formula (i.e., Pocock's sample size formula) that can be directly applied for comparison of proportions P1 and P2 in two equally sized groups. To raise the potential of the current trial, we recruited 40 patients in every group as has also been done in early studies.

#### Inclusion and exclusion criteria

*Inclusion* 18 years or older, scheduled to have coronary artery bypass grafting (CABG) and patients who were well motivated and compliant.

*Exclusion* Patients who are expected not to be able to conduct or comply with IS, such as patients with cognitive or neurological deficits, patients with coexisting acute or chronic respiratory disorders, patients unable to understand or show the proper use of the incentive spirometer, patients who cannot be instructed or supervised to assure appropriate use of the device, patients in whom cooperation is absent or patients unable to understand or demonstrate proper use of the device, patients who are confused or delirious, patients undergoing any other surgery along with CABG, having prolonged mechanical ventilation (more than 24 h) or re-intubation, patients undergoing emergency CABG surgery, chronic obstructive pulmonary disease (COPD), asthma, restrictive lung disease, preoperative major chest infection e.g. pulmonary tuberculosis, chest deformities such as pectus carinatum, pectus excavatum, thoracolumbar scoliosis, diaphragmatic hernias diagnosed on history.

**Fig. 1:**
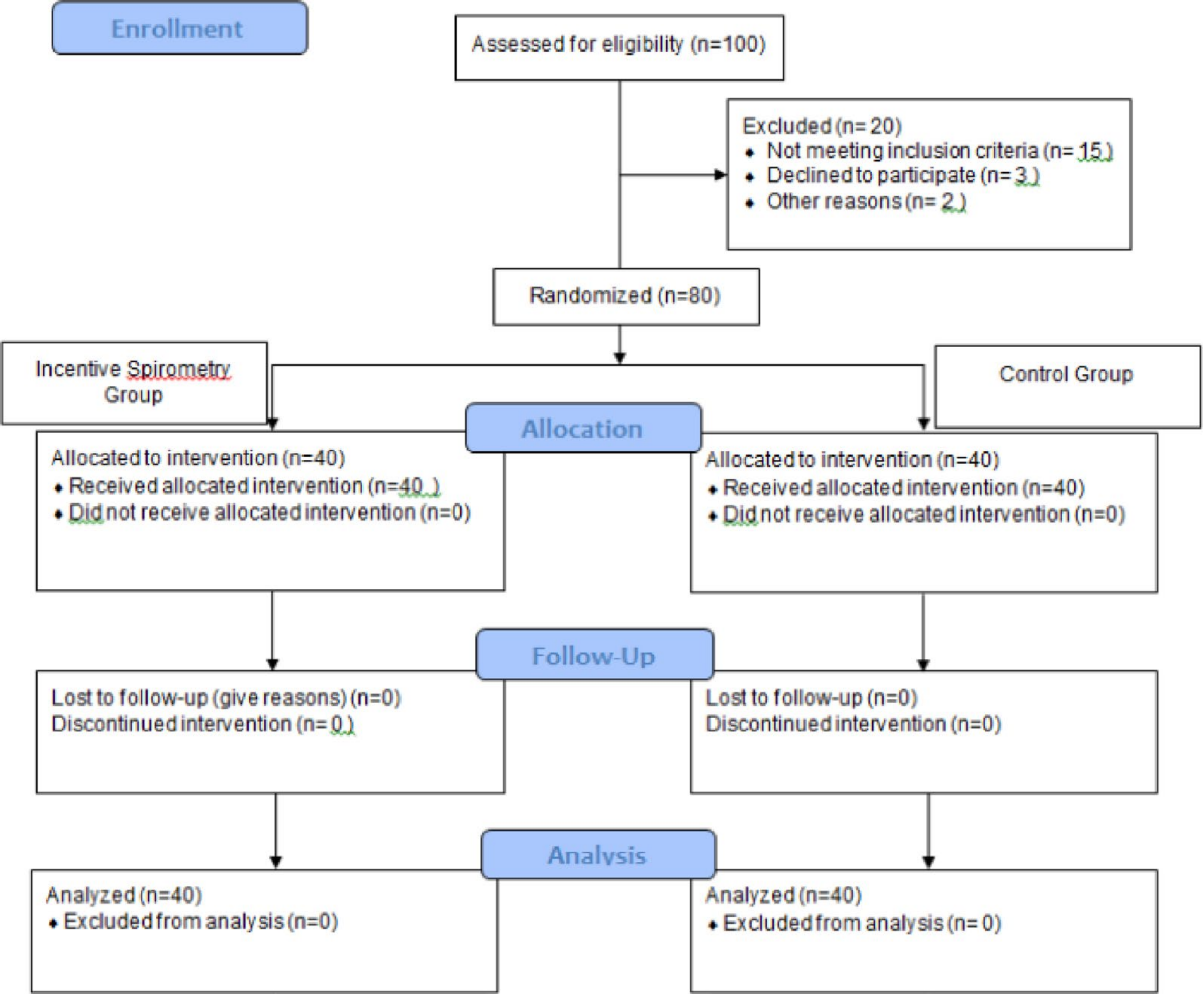
Consort diagram of patient screening and allocation

The Consort diagram of patient screening and allocation (Fig. 1) showed the patients excluded from the study in the result section. Moreover, all patients who met the criteria were involved and continued the study.

### Randomization

The participants who met the inclusion criteria were randomized into two groups according to a randomization list formatted by www.randomization.com.

Group 1: Incentive spirometry was utilized by the patient with ten breaths, six times per day for a period of 10 min in every session with a breathing technique for two days preoperatively. The patients were taught how to use IS by a nurse who would not be involved in the patient’s postoperative care. (Experimental group) (IS).

Group 2: No IS preoperatively, only IS postoperatively (Control Group).

### Blindness

The patients, health care providers included in the patient care are unaware of the treatment group allocation.

To ensure that the patients, the surgeon and the radiologist were blind to the treatment group before the study begins. The patients, surgeon, and radiologist were unaware of IS group and the surgeries performed by the same surgeon. The radiologist was also assessing the X-ray and wrote about all X-rays for all patients.

### Measured outcomes

The primary outcome was defined as respiratory complications that occur within 48–72 h following surgery, which includes: atelectasis, pneumonia, pleural effusion, and Pneumothorax, which is measured by x-ray and clinical signs and symptoms [34]. Chest X-ray was examined by the radiologist for all patients and decided if atelectasis is present or not. However, the radiologist did not mention the grade or severity of the atelectasis, but they divided them to is present if atelectasis present or not, also knowing that the radiologist was blinded about the intervention or control group, also atelectasis defined as the collapse of a part of or the entire lung, which may be acute or chronic. This research refers to the characters such as X-ray, tracheal mediastinal shift deviation; reduced respiratory activity; diminished breath sounds; displacement of the trachea to the affected side; new parenchymal thickening surrounded by hyperinflated lung [14].

Secondary outcomes included hospital length of stay that was calculated by subtracting the day of admission from the day of discharge [35]. Mechanical ventilation duration in hours, oxygenation status by measuring Pao2 and SaO2 by ABG’s, and pain control using a numerical pain scale.

### Measurement and data collection procedure

The participants who met the inclusion criteria and randomized to the intervention group were given a flow-based incentive spirometer and asked to use spirometry with deep-breathing exercise 2 days preoperatively until surgery. They were asked to hold the spirometer in the upright position, place their lips tightly across the spirometer mouthpiece, and then they were asked to slowly inhale air into the lungs to raise the ball to the target position. After that, the mouthpiece was removed, and patients were asked to hold their breath for at least 5 s, followed by normal expiration. Incentive spirometry was done with ten breaths, six times per day for a period of 10 min every session before surgery, according to literature and An-Najah National University hospital.

All patients who enrolled in the intervention were 100% compliant with preoperative IS after teaching him how to use the IS; also, reminder was used by nurses by a drug sheet that wrote by the doctors every 4 h. Also, the nurses ensured the patients' practice of IS, knowing that all patients did not know about preoperative IS tend to be healthier as what literature previously said it is no benefit.

Observations and hemodynamic parameters were measured preoperatively and postoperatively. For both groups, study observations were recorded every 6 h. Both groups postoperative received the same intervention: the exercises began on the morning after surgery with incentive spirometry, deep-breathing exercise and physiotherapy after Extubation, and early mobilization, in accordance with An-Najah University Hospital protocol. A datasheet containing the following information was filled out for each patient: name, age; height; weight; body mass index, respiratory status, medical history, presence of respiratory complications, duration of MV, and numerical pain scale and hospital length of stay.

The data collection sheet was prepared after going through the linked literature and with the supervision of experts in the field. Content validity is defined as “the degree to which objects in an instrument reflect the content universe to which the instrument will be generalized [36]. Content validity was applied while the datasheet was developed to ensure that it included all essential items [37, 38]. The assessment method for determining the validity of the data sheet included literature reviews and then follow-ups with evaluation by expert judges or panels (two intensivists, one anesthesiologist, and three nurses in critical care), and all experts’ suggestions were taken into account.

Thoracic x-rays were taken during the preoperative period and immediately following surgery in the intensive cardiac care unit (ICCU), on the ward, once the drains had been removed (48 h after surgery), and on discharge from the hospital. X-ray examinations were performed at the same frequency for all patients in both groups.

### Anaesthesia protocol

(AN-Najah National University Hospital protocol).

All patients in both groups received the same anaesthesia technique and ventilation in the operation room.

A standard induction for cardiac anaesthesia started with a facial mask inhaling sevoflurane 0–8% in 100% oxygen and fresh gas flow of 3 L/min for 5 min. After that, patients were given IV anaesthesia Propofol 2 mg/kg IV and tracheal intubation was facilitated with rocuronium 1.5–2 mg/kg with fentanyl 2–20 mcg/kg/dose initially.

### Maintenance of anaesthesia

Anaesthesia was maintained with sevoflurane 0–3% in 50% oxygen and 50% air. Neuromuscular blockade was maintained with increments of atracurium with this equation: 0.3(dose)*kg/4(concentration/ml) = ml/hr, fentanyle was used to provide intraoperative analgesia with this equation: 2(dose)*kg/20(concentration/ml) = ml/hr as 1–2 mcg/kg/hr maintenance.

For special cases like decreased ejection fraction and left main coronary disease, etomidate 0.3–0.6 mg/kg was used.

### Ethical considerations

The institutional Review Board (IRB) of An-Najah National University approved the study. Consent forms were obtained from the patients prior to participation and the study was registered in the Thai Clinical Trials Registry (No. TCTR20201020005). All patients were given both verbal and written information about the aim and objectives of the study before considering participation in the study. The study was conducted in accordance with the World Health Organization Declaration on the Ethical Principles of Helsinki for Medical Research on Humans (2013) [39].

### Analyses

The data were analysed with SPSS version 22 for Windows (IBM Corp., Armonk, NY, USA). Data normality was tested using Kolmogorov–Smirnov test. The data were not normally distributed. Thus, nonparametric statistical tests were used. The Scale data are expressed as the median (quartile 1 [Q1]–quartile 3 [Q3]). The groups were compared with the Mann–Whitney U Test. Categorical variables (YES/NO questions) were statistically analysed with Chi-square tests have been used. A *p* value ≤ of 0.05 was considered to indicate a statistically significant difference.

## Results

One hundred clients were assessed for eligibility, but 20 were excluded, 15 did not meet the inclusion criteria, three declined to participate, and two converted to PCI. The patients who did not meet the criteria switched to the hospital routine (using incentive spirometry with deep-breathing exercise postoperatively only). The remaining 80 clients were enrolled in the study and randomly allocated into two groups, as shown in the Consort diagram (Fig. 1).

### Socio-demographic and medical history characteristics of the study participants

Demographic data were comparable between the two groups (Table 1). All patients in the two groups were comparable in terms of age, gender, comorbidity, smoking, and BMI. Hemodynamic parameters and other observations were recorded before the operation, postoperatively, and three days postoperatively. There are no significant differences between the IS Group and the Control Group in all general characteristics of patients exhibited at the table 0.05 level (*p *value > 0.05).

**Table 1:** Demographic data and history

Variable	Total (Mean ± SD)	IS group (Mean ± SD)	Control group (Mean ± SD)	*p* value
Age	54.3 ± 4.5	54.4 ± 3.8	54.3 ± 5.1	0.961

Variable	Yes/no	IS group n (%)	Control group n (%)	*p* value
Gender	Male	22 (55.0%)	21 (52.5%)	0.823
	Female	18 (45.0%)	19 (47.5%)	
DM	Yes	17 (42.5%)	20 (50.0%)	0.501
	No	23 (57.5%)	20 (50%)	
HTN	Yes	15 (37.5%)	15 (37.5%)	> 0.999
	No	25 (62.5%)	25 (62.5%)	
IHD	Yes	18 (45.0%)	15 (37.5%)	0.496
	No	22 (55.0%)	25 (62.5%)	
PCI	Yes	5 (12.5%)	8 (20.0%)	0.363
	No	35 (87.5%)	32 (80.0%)	
Smoking	Yes	14 (35.0%)	16 (40.0%)	0.644
	No	26 (65.0%)	24 (60.0%)	
BMI		26.5 ± 2.6	26.4 ± 2.1	0.967
BMI category	Normal weight	12 (30.0%)	9 (22.5%)	0.727
	Overweight	24 (60.0%)	28 (70.0%)	
	Obesity	4 (10.0%)	3 (7.5%)	

### Respiratory complication

**Fig. 2:**
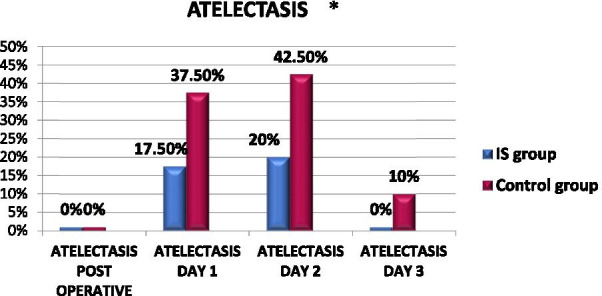
Percentage rate of atelectasis occurrence among IS and control groups. *p value of ≤ 0.05

Figure 2 shows the respiratory complications of atelectasis among the IS Group and the Control Group. On the operation day, there was no atelectasis diagnosed in either group. On the first day, of the 22 patients with atelectasis, seven were in the IS group and 15 in the Control Group, showing statistically significant differences (*p *value = 0.045). On the next day, the number of patients in the Control Group with atelectasis was 17, and in the IS group, it was 8, with statistically significant differences (*p *value = 0.030). Furthermore, the third day showed four clients with atelectasis in the Control Group and zero patients in the IS group, with statistically significant differences (*p *value = 0.040). On the other hand, there were no statistically significant differences between the groups in other respiratory complications.

### Length of stay

**Fig. 3:**
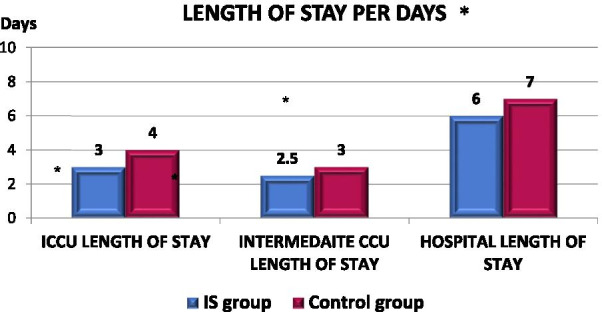
Graphical comparison of median length of stay at the hospital per day between IS and control group. *p value of ≤ 0.05

Figure 3 shows that there are significant differences between the IS Group and the Control Group in the length of stay in the intensive cardiac care unit (ICCU), intermediate cardiac care unit (IMCCU), and hospital discharge (*p *value =  < 0.001). The IS Group average was three days in ICCU, two and a half days in IMCCU. In contrast, the Control Group average was four days in ICCU and three days in IMCCU.

### Duration of mechanical ventilation

**Fig. 4:**
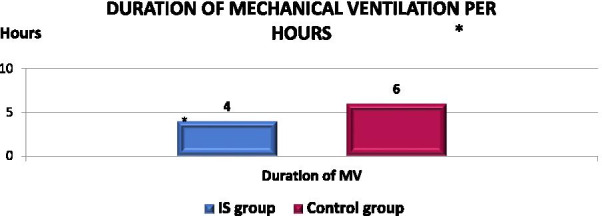
Graphical comparison of median duration of mechanical ventilation per hour between IS and control groups. *p value of ≤ 0.05

Figure 4 shows that there are significant differences between the IS Group and the Control Group in the duration of time spent in mechanical ventilation in the intensive cardiac care unit (ICCU). Patients in the IS Group spent 4 h, while patients in the Control Group spent 6 h (*p *value =  < 0.001). The median hours spent was 5 h. However, the incidence of re-intubation was (0.0%) with (*p *value =  > 0.999).

### Oxygenation

#### The partial pressure of oxygen (Pao2)

**Fig. 5:**
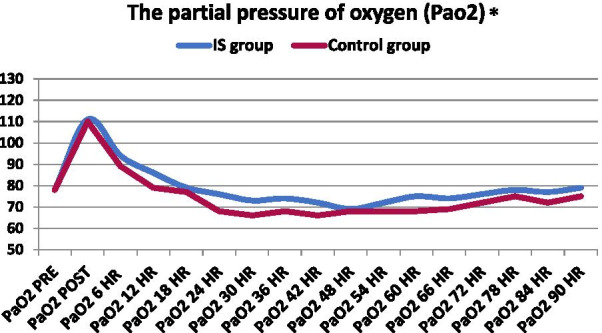
Graphical comparison of median partial pressure of oxygen (Pao2) between IS and control groups. *p value of ≤ 0.05

Figure 5 shows significant differences between the IS Group and the Control Group in the partial pressure of oxygen (Pao2), with obvious improvement in Pao2 in the IS Group, as shown in the *p* value from 6 to 90 h. On the other hand, there were no significant differences between the groups pre-and postoperatively with (*p *value = 0.900 and 0.149), respectively.

#### Oxygen saturation of arterial blood (SaO2%)

**Fig. 6:**
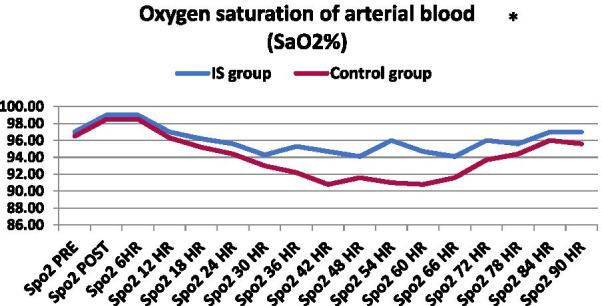
Graphical comparison of median oxygen saturation of arterial blood (SaO2%) between IS and control groups. *p value of ≤ 0.05

Figure 6 shows that there are significant differences between the IS Group and the Control Group in the oxygen saturation of arterial blood (Sao2), with obvious differences, as shown in the *p *value in all postoperative measurement times. On the other hand, there were no significant differences between the preoperatively groups (*p *value = 0.335).

#### Numerical rating scales (NRS) pain scale

**Fig. 7:**
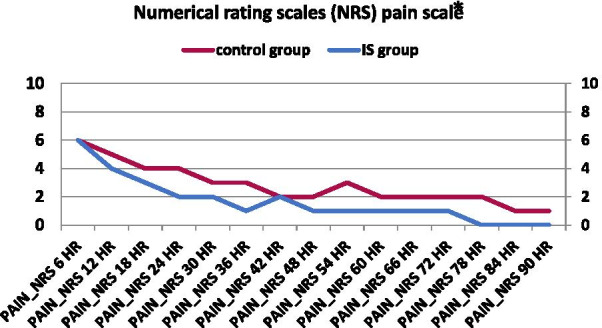
Graphical comparison of the median numerical rating scale (NRS) pain scale between IS and control groups. *p value of ≤ 0.05

Figure 7 shows that there were significant differences between the IS Group and the Control Group in the numerical rating scale (NRS) pain scale, with obvious less pain in the IS Group than the control when using the same analgesic plan, as shown in the *p *value below in all measurements at all times except at 12 h. In the NRS, scores ≤ 5 corresponded to mild, 6–7 to moderate, and ≥ 8 corresponded to severe pain.

## Discussion

The results of this study indicate that IS was used preoperatively for patients with CABG surgery to reduce postoperative atelectasis, length of hospital stay, and improved postoperative oxygenation.

### The effect of preoperative incentive spirometry on atelectasis

In the current study, there was a significant decrease in the incidence of atelectasis in the IS Group. This finding is consistent with Diken and Özyalçın [31], who conducted an RCT that involved 108 patients divided into two groups: IS preoperative and routine care for control, with a body mass index over 30 kg/m^2^ and without previous pulmonary disease. In Diken and Özyalçın’s study, patients with atelectasis were predominantly higher in the Control Group compared to the IS Group (18 vs. 7 patients, respectively) (*p* = 0.0036). The current findings also agree with Gilani et al. [40], who conducted an RCT that showed the incidence of postoperative atelectasis was 14.10% in Group I (IS) and 27.10% in Group II patients (control) (*p* = 0.04). Moreover, the current study results are consistent with Oshvandi et al. [11], who was showed that the occurrence of atelectasis, respiratory status, dyspnea, and sweating showed a significant difference between the IS and control groups at all hours after surgery (*p* < 0.001). Furthermore, the results are also in agreement with Shaban et al. [10], who showed the incidence rate of atelectasis in the experimental group was (26.7%), less than the control group (56.7%) with (*p* = 0.01). In addition, the study results also agree with Nardi et al. [19], who revealed that better clinical results for respiratory and musculoskeletal function were found in the groups preoperatively treated with physiotherapeutic protocols immediately before as well as after cardiac surgery. Just the same results were confirmed by Yánez-Brage et al. [12] in their observational study, which was conducted on 263 patients and revealed that preoperative physiotherapy (involving incentive spirometry, deep-breathing exercises, assisted coughing and early ambulation) after off-pump CABG surgery was related to a lower incidence of atelectasis.

Conversely, the current study is inconsistent with a study conducted by Moradian et al. [32]. This study examined 100 participants and found no significant differences between the IS and control groups in terms of atelectasis and hypoxemia (*p *value > 0.05). Freitas et al.[13] also revealed no evidence of a difference between groups in the incidence of PPCs with IS and treatment with physical therapy, positive-pressure breathing techniques, active cycle of breathing, or preoperative patient education and worse pulmonary function and arterial oxygenation. Eltorai et al. [25] investigated the clinical effectiveness of incentive spirometry and found that there was narrow evidence to support its advantages and an absence of a harmonized protocols for its use. In addition, Overend et al. [21] conducted a systematic review study and concluded that evidence does not support the use of IS for decreasing the incidence of PPCs.

### The effect of preoperative incentive spirometry on oxygenation

The current results showed a significant improvement in Pao2, Sao2, and SPO2 in the IS Group compared to the Control Group. These results are consistent with Fayyaz et al. [28], where preoperative spirometry had improved postoperative oxygenation in the IS group to 97.29 while the control group was 93.27. These results are in line with Yazdemik et al. [29], who concluded that incentive spirometry caused a remarkable improvement of Pao2, Sao2, and SPO2. Another study conducted by Moradyan et al. [30] corresponds to the current study results and revealed that protocols for breathing exercises (deep breathing, incentive spirometry, and directed maneuvers) could improve PaO2 and SaO2. In contrast, Balandiuk and Kozlov [33] revealed that the use of an incentive spirometer preoperatively for cardiac surgery significantly improved arterial oxygenation.

Freitas et al. [13] presented results that contradict the current results. They found no differences between the study groups in terms of incentive spirometry used and found poorer lung function and status of arterial oxygenation competed with those treated with positive pressure ventilation. Diken and Özyalçın [31] also disagree with our study results after conducting RCT with the two groups and showed no change in oxygenation status for both groups. Even Afrasiabi et al. [24] reported that incentive spirometry had no significant effect on the improvement in postoperative oxygenation. In addition, Yánez-Brage et al. [12] also reported that the improvement in postoperative oxygenation using incentive spirometry is not permanent; this improvement is reversible after a short period of time. Carvalho et al. [20] in a systematic review study, reviewed 30 studies in relation to IS. They reported that there was no strong evidence to support the use of IS after CABG, and there is a need for studies to clarify the effect and justify the use of this technique.

### The effect of preoperative incentive spirometry on hospital length of stay

The current results showed that the incidence of hospital length of stay for the IS Group was 6 days, while in the Control Group it was 7 days. ICU LOS for the IS Group was also reduced compared with the Control Group. This finding is consistent with Nardi et al. [19] who revealed that the hospital stay was further reduced in the IS group. In addition, Shaban et al. [10] reported the same results when they declared that the hospital length of stay decreased for the IS group compared to the control group. On the other hand, Fayyaz et al. [28] presented results that contradict the current results. They revealed that there were no differences between the groups in length of hospital stay.

### The effect of preoperative incentive spirometry on mechanical ventilation time

The current results showed significant differences between the two groups (IS and Control) regarding mechanical ventilation time (duration), which was 4 h for the IS Group and 6 h for the Control Group. This finding is consistent with Gilani et al. [40], who showed that mechanical ventilation time was significantly less in Group I patients (IS): it was 5.49 + 2.28 h versus 6.74 + 5.46 h in Group II patients (control) (p value 0.05). This finding also agrees with Balandiuk and Kozlov [33], who reported that a significant decrease in the duration of MV in the IS group was 7.3 h compared to 10.4 h (*p* < 0.05) in the control group.

Moradian et al. [32] presented results that contradict the current study results. They revealed that there were no differences between the groups in mechanical ventilation time, with 10.5 h for the IS group and 11.5 h for the control group. Yazdemik et al. [29] also reported the same duration of mechanical ventilation in both groups following coronary artery bypass surgery, which means there were no differences between the groups. Moreover, Afrasiabi et al. [24] presented results that contradict the current study results. They found no differences in mechanical ventilation time between the study groups. Furthermore, Yánez-Brage et al. [12] in an observational study, showed that no statistical differences were observed during the time of mechanical ventilation between the study groups.

### The effect of preoperative incentive spirometry on pain control

The current results showed significant differences between the incentive spirometry group and the control group in the numerical rating scale (NRS) pain scale with obvious pain in the IS group rather than control using the same analgesic plan as shown *p *value in all measurements at all times except at 12 h. To consider NRS, scores ≤ 5 correspond to mild, 6–7 to moderate, and ≥ 8 to severe pain. However, deep breathing exercises and cough may cause pain to patients, but patients well educated about incentive spirometer preoperative will be less needed to analgesia and better improvements in respiratory status. This finding is agreed with Renault et al. [9], who report that pain and postoperative fear are associated with changes in lung mechanics that affect the performance of periodic deep inspiration and effective cough with effective spirometry, allowing the accumulation of secretion, alveolar collapse, and changes in gas exchange, early incentive to cough decreases pain and better control.

### IS the clinical application, and what is new?

The current study demonstrated the clinical application and IS a protocol that showed important results in reducing atelectasis occurrence in CABG patients. Meanwhile, some of the clinical trials question the effectiveness of IS use and why it is still prescribed to patients in different locations, especially after cardiac surgery [22, 25]. This paper suggests the implication of this protocol in intensive cardiac care units, especially with critical care nurses.

Proper preoperative incentive spirometry concurrent with deep breathing exercises and coughing every 4 h as a new protocol will enhance and improve the respiratory status, especially atelectasis with others improving in patient’s status that leads to a better outcome and less pain, however, patients covered with analgesics, but less use it.

### Recommendations

This study was performed on patients who received invective spirometry for two days preoperative and did not have lung problems. Therefore, it is recommended to conduct a clinical study to examine incentive spirometry with deep breathing and cough trials in patients who will undergo CABG surgery with lung problems such as chronic obstructive pulmonary disease and asthma.

## Conclusions

Preoperative incentive spirometry, along with deep breathing exercises, assisted coughing, and early ambulation after coronary bypass surgery is related to the prevention and lower incidence of atelectasis, hospital length of stay, duration of mechanical ventilation, and improved postoperative oxygenation. A difference that can be considered both significant and clinically relevant.

## Data Availability

Data used to support the findings of this study is presented in the manuscript or available from the corresponding author (Essa M Sweity, E-mail: E.sweity@najah.edu) upon reasonable request.

## Abbreviations

IS: Incentive spirometry
POLS: Postoperative hospital length of stay
NNUH: An Najah National University Hospital
PPCs: Postoperative pulmonary complications
CABG: Coronary artery bypass graft
WHO: World Health Organization
IRB: Institutional review board
SPSS: Statistical Package for the Social Sciences
CAD: Coronary artery disease
IPPB: Intermittent positive pressure breathing
PaO2: Partial pressure of oxygen
Sao2: Invasive oxygen saturation
Spo2: Non- Invasive oxygen saturation
PaCo2: Partial pressure of carbon dioxide
ICU: Intensive Care Unit
ECG: Electrocardiogram
RCT: Randomized control trial
MV: Mechanical ventilator
ABG’s: Arterial blood gases
OR: Operation room
IMCCU: Intermediate cardiac care unit
NRS: Numerical rating scale

## References

[1] WHO. The top 10 causes of death. Fact sheets 2018. http://www.who.int/news-room/fact-sheets/detail/the-top-10-causes-of-death.

[2] Hillis LD, Smith PK, Anderson JL, Bittl JA, Bridges CR, Byrne JG, Cigarroa JE, DiSesa VJ, Hiratzka LF, Hutter AM (2011). 2011 ACCF/AHA guideline for coronary artery bypass graft surgery: executive summary: a report of the American College of Cardiology Foundation/American Heart Association Task Force on Practice Guidelines developed in collaboration with the American Association for Thoracic Surgery, Society of Cardiovascular Anesthesiologists, and Society of Thoracic Surgeons. J Am Coll Cardiol.

[3] Branson RD (2013). The scientific basis for postoperative respiratory care. Respir Care.

[4] Mullen-Fortino M, O'Brien N, Jones M (2009). Critical care of a patient after CABG surgery. Nurs2020 Crit Care.

[5] Miskovic A, Lumb AB (2017). Postoperative pulmonary complications. Br J Anaesth.

[6] Hulzebos EH, Helders PJ, FaviÚ NJ, De Bie RA, de la Riviere AB, Van Meeteren NL (2006). Preoperative intensive inspiratory muscle training to prevent postoperative pulmonary complications in high-risk patients undergoing CABG surgery: a randomized clinical trial. JAMA.

[7] Ferreira GM, Haeffner MP, Barreto SSM, Dall'Ago P (2010). Espirometria de incentivo com pressão positiva expiratória é benéfica após revascularização miocardio. Arquivos Brasileiros de Cardiologia.

[8] Massard G, Wihlm JM. Postoperative atelectasis. Chest Surg Clin N Am. 1998;8(3):503–528, viii. 9742334

[9] Renault JA, Costa-Val R, Rosseti MB, Houri Neto M (2009). Comparação entre exercícios de respiração profunda e espirometria de incentivo no pós-operatório de cirurgia de revascularização do miocárdio. Braz J Cardiovasc Surg.

[10] Shaban M, Salsali M, Kamali P, Poormirzakalhori R (2002). Assessment the effects of respiratory exercise education in acute respiratory complication and the length of patient hospitalization, for undergoing coronary artery bypass surgery in Kermanshah Emam Ali Hospital. J Hayat.

[11] Oshvandi K, Bostanbakhsh A, Salavati M, Bakhsai M, Moghimbeighi A, Maghsoudi Z (2020). Effect of respiratory exercises on the prevalence of atelectasis in patients undergoing coronary artery bypass surgery. Avicenna J Nurs Midwifery Care.

[12] Yánez-Brage I, Pita-Fernández S, Juffé-Stein A, Martínez-González U, Pértega-Díaz S, Mauleón-García Á (2009). Respiratory physiotherapy and incidence of pulmonary complications in off-pump coronary artery bypass graft surgery: an observational follow-up study. BMC Pulm Med.

[13] Freitas E, et al. Incentive spirometry for preventing pulmonary complications after coronary artery bypass graft. Cochrane Database Syst Rev. 2012;3(9):Cd004466. 10.1002/14651858.CD004466.pub3 PMC8094624 22972072

[14] David A Grooms MSHS R. Postoperative pulmonary complications. Clin Found. 2012;13:1–11.

[15] Wynne R, Botti M (2004). Postoperative pulmonary dysfunction in adults after cardiac surgery with cardiopulmonary bypass: clinical significance and implications for practice. Am J Crit Care.

[16] Zarbock A, Mueller E, Netzer S, Gabriel A, Feindt P, Kindgen-Milles D (2009). Prophylactic nasal continuous positive airway pressure following cardiac surgery protects from postoperative pulmonary complications: a prospective, randomized, controlled trial in 500 patients. Chest.

[17] Westwood K, et al. Incentive spirometry decreases respiratory complications following major abdominal surgery. Surgeon. 2008;5(6):339–42. 10.1016/s1479-666x(07)80086-2 18080608

[18] Anandhi D, Divya P (2018). Influence of various factors on the incentive spirometry values in patients undergoing thoracotomy. Ann Physiother Clin.

[19] Nardi P, Pellegrino A, Pisano C, Vacirca SR, Anselmi D, Saulle S, Dandi R, Romano A, Servadio A, Gianlorenzi A (2019). The effect of preoperative respiratory physiotherapy and motor exercise in patients undergoing elective cardiac surgery: short-term results. Kardiochirurgia i torakochirurgia polska = Pol J Cardio Thorac Surg.

[20] Carvalho CRF, Paisani DM, Lunardi AC (2011). Incentive spirometry in major surgeries: a systematic review. Braz J Phys Ther.

[21] Overend TJ, Anderson CM, Lucy SD, Bhatia C, Jonsson BI, Timmermans C (2001). The effect of incentive spirometry on postoperative pulmonary complications: a systematic review. Chest.

[22] Restrepo RD, Wettstein R, Wittnebel L, Tracy M (2011). Incentive spirometry: 2011. Respir Care.

[23] Ünver S, Kıvanç G, Alptekin HM (2018). Deep breathing exercise education receiving and performing status of patients undergoing abdominal surgery. Int J Health Sci.

[24] Afrasiabi A, Ansarin KH, Salmasi S (2006). Comparison of the effect of incentive spirometry on pulmonary volumes and arterial blood gases after coronary artery bypass surgery. Armaghane Danesh.

[25] Eltorai AEM, Baird GL, Eltorai AS, Pangborn J, Antoci V, Cullen HA, Paquette K, Connors K, Barbaria J, Smeals KJ (2018). Perspectives on incentive spirometry utility and patient protocols. Respir Care.

[26] Agostini P, Singh S (2009). Incentive spirometry following thoracic surgery: what should we be doing?. Physiotherapy.

[27] Gürlek Ö, Yavuz M (2013). CERRAHİ KLİNİKLERDE ÇALIŞAN HEMŞİRELERİN AMELİYAT ÖNCESİ HASTA EĞİTİMİ UYGULAMA DURUMLARI. Anadolu Hemşirelik ve Sağlık Bilimleri Dergisi.

[28] Fayyaz F, Ammar A (2016). Preoperative incentive spirometry; effectiveness to improve postoperative oxygenation in patients undergoing CABG surgery. Prof Med J.

[29] Yazdannik A, Bollbanabad HM, Mirmohammadsadeghi M, Khalifezade A (2016). The effect of incentive spirometry on arterial blood gases after coronary artery bypass surgery (CABG). Iran J Nurs Midwifery Res.

[30] Moradyan T, Farahani M, Mohammadi N, Jamshidi R (2012). The effect of planned breathing exercises on oxygenation in patients after coronary artery bypass surgery. Iran J Cardiovasc Nurs.

[31] Diken ÖE, Özyalçın S (2018). OP-348—preoperative incentive spirometry exercise reduces the risk of atelectasis in Obese Cabg patients. Am J Cardiol.

[32] Moradian ST, Heydari AA, Mahmoudi H (2019). What is the role of preoperative breathing exercises in reducing postoperative atelectasis after CABG?. Rev Recent Clin Trials.

[33] Balandiuk AE, Kozlov IA (2004). Incentive spirometry for preoperative preparation of cardiac patients: 036. Eur J Anaesthesiol: EJA.

[34] Miskovic A, Lumb AB (2017). Postoperative pulmonary complications. Br J Anaesth: BJA.

[35] Carter EM, Potts HW (2014). Predicting length of stay from an electronic patient record system: a primary total knee replacement example. BMC Med Inform Decis Mak.

[36] Straub D, Boudreau M-C, Gefen D (2004). Validation guidelines for IS positivist research. Commun Assoc Inf Syst.

[37] Boudreau M-C, Gefen D, Straub DW (2001). Validation in information systems research: a state-of-the-art assessment. MIS Q.

[38] Lewis BR, Snyder CA, Rainer RK (1995). An empirical assessment of the information resource management construct. J Manag Inf Syst.

[39] Association WM (2013). World Medical Association Declaration of Helsinki: Ethical principles for medical research involving human subjects. JAMA.

[40] Gilani SRA, Hussain G, Ahmad N, Baig MAR, Zaman H. Comparison of postoperative atelectasis in patients undergoing coronary artery bypass grafting with and without preoperative incentive spirometry. J Postgrad Med Inst (Peshawar-Pakistan). 2016;30(2):169–72.

